# Not All Is Lost: Old Adults Retain Flexibility in Motor Behaviour during Sit-to-Stand

**DOI:** 10.1371/journal.pone.0077760

**Published:** 2013-10-25

**Authors:** Christian Greve, Wiebren Zijlstra, Tibor Hortobágyi, Raoul M. Bongers

**Affiliations:** 1 University of Groningen, University Medical Center Groningen, Center for Human Movement Science, Groningen, The Netherlands; 2 Institute of Movement and Sports Gerontology, German Sport University, Cologne, Germany; Katholieke Universiteit Leuven, Belgium

## Abstract

Sit-to-stand is a fundamental activity of daily living, which becomes increasingly difficult with advancing age. Due to severe loss of leg strength old adults are required to change the way they rise from a chair and maintain stability. Here we examine whether old compared to young adults differently prioritize task-important performance variables and whether there are age-related differences in the use of available motor flexibility. We applied the uncontrolled manifold analysis to decompose trial-to-trial variability in joint kinematics into variability that stabilizes and destabilizes task-important performance variables. Comparing the amount of variability stabilizing and destabilizing task-important variables enabled us to identify the variable of primary importance for the task. We measured maximal isometric voluntary force of three muscle groups in the right leg. Independent of age and muscle strength, old and young adults similarly prioritized stability of the ground reaction force vector during sit-to-stand. Old compared to young adults employed greater motor flexibility, stabilizing ground reaction forces during sit-to-sand. We concluded that freeing those degrees of freedom that stabilize task-important variables is a strategy used by the aging neuromuscular system to compensate for strength deficits.

## Introduction

Sit-to-stand is a fundamental activity of daily living performed up to 70 times a day [1], [2]. Yet over 60% of nursing home residents report difficulty in transferring in and out of a chair and bed [3]. Successful and safe completion of the sit-to-stand task requires sufficient leg strength and adequate coordination of multiple body segments [4], [5]. A critical element of this coordination is the transfer of trunk momentum generated just after lift-off into anterior-posterior and vertical movements of the center of mass (CoM) coupled with properly scaled and timed ground reaction forces (GRF) [5], [6]. It is reasonable to expect that the age-related ∼50% decline in maximal voluntary force in hip, knee, and ankle muscles affects motor coordination during sit-to-stand [7], [8]. Due to the decline in maximal leg strength, healthy old compared to young adults use twice as much of the available leg strength and operate at 80-100% of maximum muscular capacity [9], [10]. This high physiological demand forces old compared with young adults to adjust the way they stand up from a chair and seek stability. Old adults generate larger trunk flexion just before lift-off, decrease peak GRF, and impart less of the propulsive power to the CoM [4], [11]–[14]. The present paper aims to establish whether these adaptations affect old adults in their choice of the primary performance variable and whether there are age-related differences in flexibility of motor behaviour during the sit-to-stand task.

In the current study we explored the idea that old compared to young adults use different motor coordination strategies during the sit-to-stand task as a compensatory mechanism for physical impairments [4], [12], [13], [15]. The function of this altered coordination would be to increase stability of task-important performance variables, a concept proposed previously in conjunction with the sit-to-stand task but not tested in old adults [16], [17]. Using the uncontrolled manifold (UCM) analyses, these authors suggested that the CoM position [16], [18] and momentum [17], [19] were the critical kinematic and kinetic variables stabilized by the neuromuscular system during sit-to-stand. Here we extend these findings and apply, to our knowledge for the first time, a comparative UCM analysis of the sit-to-stand task between young and old adults. Based on previous findings and the kinematic and kinetic events during sit-to-stand we chose to examine the CoM, head position, GRF and linear and angular momentum of the CoM as performance variables [6], [16]–[19].

The UCM analysis, as compared to other measures such as pair-wise correlation or bivariate covariance analysis, enables to study motor coordination patterns that involve multiple degrees of freedom (DOF) [20], [21]. This feature is essential when studying motor coordination patterns during functional tasks involving multiple body segments such as sit-to-stand. Within the UCM analysis it is assumed that the neuromuscular system acts in a state space of elemental variables (e.g. joint angles) and makes use of all available DOF to enable stable but flexible control of task-important performance variables (e.g. CoM) [21]. Accordingly, numerous degrees of freedom form an advantage for the neuromuscular system during accurate performance of motor tasks which is known as the “principle of motor abundance” [21], [22]. Elemental variables are defined as those degrees of freedom that can be changed independent of each other [21]. Performance variables are those variables that the neuromuscular system controls to achieve successful execution of a motor task [18], [21]. As detailed elsewhere, the UCM analysis decomposes trial-to-trial variability in elemental variables into variability within the uncontrolled manifold (V_UCM_) and variability deviating from this uncontrolled manifold (V_ORT_) [18], [21], [23]. V_UCM_ quantifies the extent to which elemental variables co-vary to stabilize a performance variable around its mean. V_ORT_ represents the extent to which elemental variables destabilize a performance variable away from its mean position. The value of the ratio V_UCM_/V_ORT_ (V_RATIO_) indicates to what extent the neuromuscular system makes use of motor abundance to stabilize a performance variable and identifies the performance variable of primary importance [18], [21]. Of particular relevance of the UCM analysis to the present study is its ability to detect age-related changes in the flexibility of the motor behaviour [21], [24]–[27]. Therefore, the purpose of the current study was to establish the performance variable of primary importance and whether old differed from young adults in the flexibility of their motor behaviour as they perform the sit-to-stand task.

Based on data from previous studies, the emerging hypothesis is that young and old adults most likely prioritize stability of different performance variables due to the well-characterized age-related differences in neural, musculoskeletal, and physiological aspects [7]–[10], [28]. Thus we hypothesize that: 1) the sagittal plane kinematics of the CoM is the performance variable of primary importance in young adults [18], [29]; 2) the GRF vector is the performance variable of primary importance in old adults [6], [8], [14]. Concerning the age-related differences in the flexibility of motor behaviour we refer to two competing ideas: The first idea is based on several studies using UCM analyses which reported that old compared with young adults employ a less flexible motor behaviour during a variety of motor tasks [24]–[27], [30]. These findings suggest that motor flexibility might also be poorer in old adults during sit-to-stand tasks. The second idea is based on the notion that in these previous studies task demand was low and similar for young and old adults [24]–[26], [30]. Considering that even healthy old adults perform the sit-to-stand task at 80–100% of maximum knee moment [9], [10], the possibility exists that, unlike in low demanding tasks, flexibility in motor behaviour increases in compensation for muscular strength deficits. A similar concept has been proposed in previous studies, which documented that healthy young adults employ a more flexible motor behaviour when standing up under more challenging task conditions [16], [17]. Based on these two notions we hypothesize, 3) that old compared with young adults differ in their flexibility of motor behaviour but based on the literature we cannot predict a direction of this difference.

## Methods

### Participants

In total 15 healthy young (23.8±2.2 years; 8 males and 7 females) and 11 old (76±5.1 years; 6 males and 5 females) adults participated in the study. Participants were excluded from the experiment when they suffered any neurological disease affecting motor function, arm or leg pain, musculoskeletal impairments, other than strength deficits, affecting sit-to-stand performance, fear of falling, and a fall during sit-to-stand in the last six months. To be included, participants had to be able to consecutively rise 25 times from a chair set at 100% of each subject’s lower leg length.

### Ethics Statement

The ethics committee in the Center for Human Movement Sciences, University Medical Center Groningen approved the study that was conducted according to the principles expressed in the Declaration of Helsinki. Before the start of the study, each participant read and signed a written informed consent.

### Experimental set-up

This study focused on the sagittal plane analysis of the sit-to-stand task. We collected data with an Optotrak motion capture system consisting of two cameras and a Kistler force platform. The two systems were synchronized through an analog-to-digital converter that sampled the data at 100 Hz. 11 LEDs were placed on the participants’ right side: on the base of 5th metatarsal, 2 cm inferior to lateral malleolus, lateral femoral epicondyle, greater femoral trochanter, inferior to lateral aspect of acromion process, lateral humeral epicondyle just superior to radiohumeral junction, styloid process of radius, immediately anterior to external auditory meatus, skin of left pelvis approximately 20% of distance from greater trochanter to shoulder and one-third of the distance from posterior to anterior iliac spine (L5/S1 junction), posterior trunk at thoracic vertebra 12 and cervical vertebra 7.

### Experimental procedure

#### Isometric strength profiles

At the start of the experiment each participant’s maximal isometric strength on the right side was measured in the following muscle groups using a handheld dynamometer as detailed previously [31], [32]: ankle dorsiflexor, knee extensors and flexors, and hip extensors and flexors. Symmetry in leg strength between right and left side was assumed. Participants warmed up by performing three contractions for each muscle group at 50–60% of maximum followed by three maximum effort isometric contractions for 4 s with each muscle group. There was 10 s rest between the warm-up trials and 30 s of rest between the maximum effort isometric contractions. Participants did not report fatigue. The mean of the maximum effort isometric contractions for each muscle was used in the analysis.

#### Sit-to-stand task

Participants sat on a chair without armrests. Chair height was set at 100% of each subject’s lower leg length using the fibula head as a reference point. Participants were instructed to sit upright and place their hands on the thighs and both feet on the force plate in front of the chair. Feet were placed symmetrically next to each other at shoulder width. The starting position of the trunk, head, arm, leg, and feet placement was standardized for each individual participant and checked, and corrected as needed, before each trial.

After a verbal “GO” signal, participants rose at a self-chosen comfortable speed. They were free to reposition their arms but were not allowed to push with their hands on the thighs or swing the right arm. Once upright, participants remained in that position for 2 s. The analysis focused on the rising phase of the sit-to-stand task. Each participant performed 25 sit-to-stand trials. There was 5 s of rest after each trial, and, if the participant needed, 1 min of rest after 10 trials.

### Data analysis

Coordinate data of each marker and force plate data were filtered using a bi-directional 4^th^ order low-pass Butterworth filter with a cut-off frequency of 6 Hz. Marker coordinates were processed to calculate joint angles and angular velocities of the foot, ankle, knee, hip, trunk, head, shoulder, elbow and wrist in the sagittal plane. Customized data analysis programs were run in MATLAB R2012. Duration of each sit-to-stand trial was determined by the initiation of forward trunk movement and end of trunk motion, defined by a threshold of angular change of .009 radians within 5 ms. Accuracy of the algorithm in event detection was visually controlled for each sit-to-stand trial. Lift-off was determined as the point in time at which a directional change of the trunk motion from flexion to extension occurred [33]. Based on event detection of the initiation and end of the sit-to-stand movement each sit-to-stand trial was time-normalized. The variability components V_UCM_, V_ORT_ and V_RATIO_ (V_UCM_/V_ORT_) of the time normalized sit-to-stand trials were partitioned into three phases (preparatory phase (1–30%), lift-off (31–60%) and extension phase (61–100%)) and averaged across these phases for all performance variables. Data analysis and interpretation of the results focused on the lift-off and extension phase of the chair rise.

### Mechanical demands

We performed a 2D analysis and through linear and angular Newtonian equations computed knee and hip joint moment [34]. The peak knee and hip extensor moments were normalized by body mass. The CoM and moment of inertia of each segment were calculated based on mass and sex of each subject [34]. We used these data to quantify the age-related differences in mechanical demand during the sit-to-stand task.

### Performance variables

#### Sagittal plane CoM

The location of the whole body CoM was calculated based on the participants body-segment lengths and the estimated locations and proportions of segmental masses [34]. The sagittal plane CoM position was calculated by 8 segmental angles with the horizontal (foot, shank, thigh, trunk, upper arm (ua) and lower arm (la)). The CoM position in the sagittal plane was calculated by using equation 1:
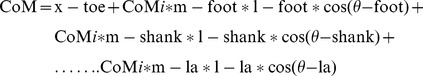
(1)


Where x-toe is the position of the foot in the anterior-posterior-direction, CoMi the estimated locations of the CoM on the ith segment, m the proportion of total body mass of each segment, l the length of the segment and θ the segmental angle. Grand means of segmental length based on all trials to be representative of a constant segmental length were used.

#### Vertical and anterior-posterior ground reaction force

Vertical and anterior-posterior GRF data were derived from the Kistler force-plate. Analyzed peak vertical GRF data were normalized by body weight in kg.

#### Linear and angular CoM momentum

The linear CoM momentum was calculated using equation 2: 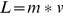
(2)


Where m is the mass of the subject in kilograms and v is the velocity of the CoM of the body in meters per second.

Angular momentum of the CoM was calculated using equation 3:
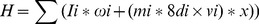
(3)


Where mi is the mass of the ith segment, 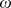
i the angular velocity of the ith segment, Ii the moment of inertia of the ith segment, di the vector from the ith segment CoM relative to the total body CoM and vi the velocity of the ith segment CoM relative to the velocity of the total body CoM.

#### Head position

Head position in space was defined by the coordinates of the marker positions immediately anterior to the external auditory meatus.

### Mean phase standard deviation of performance variables

To determine age-related differences in variability of performance variables across trials, we computed standard deviations of performance variables at each percentage of the movement trajectory. The standard deviations were then averaged across the phases (1–30%, 31–60% and 61–100%) of the sit-to-stand movement. However, if consistency of performance variables underlies multi-joint coordination patterns was established by comparing the different UCM components (V_UCM_ and V_ORT_) with respect to the UCM of each performance variable [18].

### UCM analysis

Analyzing motor tasks with the UCM approach follows the execution of the following steps [18], [21]: 1.) Selection of elemental variables: Depending on the motor task under scrutiny different elemental variables can be chosen to define the system’s state space and analyzed whether they flexibly stabilize a hypothesized performance variable; 2.) Selection of performance variables: A variable which is affected by changes of a set of chosen elemental variables can be selected as performance variable for the analysis of a motor task; 3.) Creating a linear model of the system: Relations between small changes in elemental variables and the selected performance variables are computed and united in a Jacobian matrix. After computation of the Jacobian, its null-space is used as a linear approximation of the UCM; 4.) Partitioning variance into V_UCM_ and V_ORT_: A hypothesis about a variable being a controlled variable is supported if V_UCM_ is higher than V_ORT_, that is the ratio V_UCM_/V_ORT_ (V_RATIO_) is larger than 1.

#### Selection of elemental and performance variables

Elemental variables were sagittal plane joint angles and angular velocities of the foot, ankle, knee, hip, trunk, head, shoulder and elbow. Performance variables were the sagittal plane anterior-posterior and vertical CoM, head and GRF and the anterior-posterior, vertical and angular CoM momentum. Accordingly for the calculation of the CoM, head and GRF the elemental variables consisted of eight degrees of freedom and for the calculation of the CoM momentum the elemental variables consisted of 16 degrees of freedom. Note that analyzing the anterior-posterior and vertical dimensions of the performance variables separately does not mean that those dimensions are controlled independently by the neuromuscular system [18]. However, a previous study suggested to analyze these dimensions separately in order not to miss potential significant stabilizing effects produced by each dimension [18].

#### Creating a linear model of the system

In order to relate changes in elemental variables to changes in performance variables, it is necessary to obtain the geometrical models linking the position of the performance variable to the state space configurations. However, recent UCM studies suggested that multiple linear regression (MLR) analysis might also be a valid tool for computing the Jacobian [35], [36]. Freitas et al (2010) showed that MLR is valid for calculating the Jacobian of the sagittal plane CoM and anterior-posterior center of pressure with three DOF in healthy persons during a standing balance task [36]. However, it is not established yet if MLR is a valid method in an eight DOF analysis in young and old adults during a sit-to-stand task. Therefore we examined whether calculation of the Jacobian by MLR was valid for the anterior-posterior CoM in an eight DOF system. We thus compared the results from the traditional geometric model approach with the results from the MLR analysis. The online supplements show the geometric model of the CoM and methodological details, results, validation, and discussion on the MLR analysis (Methods S1, Results S1 and Discussion S1). We demonstrated that there was no statistically significant difference between the two approaches; hence, we used MLR for the UCM analysis.

### Partitioning Variance into V_UCM_ and V_ORT_

The decomposition of variability in the state space of elemental variables was based on 25 chair rise trials (N). Acquired values of all performance variables were time-normalized with a cubic spline interpolation and partitioned into 100 equidistant time intervals. For each time interval, we computed the mean state space configuration (M(t)) across trials. For each particular time interval, the corresponding state space configuration (A_k_(t)) was subtracted from the M(t) obtaining Δk(t) [M(t) – A_k_(t)]. Delta k (Δk) represents the variance of the joint configuration of the k_th_ trial from the mean joint configuration at each time interval (t). Next, the elemental variables of the state space configuration were decomposed into two components, variance along the UCM (Δk_UCM_) and variance orthogonal to the UCM (Δk_ORT_) [18], [23]. The UCM was the null-space of the Jacobian. The null space of the Jacobian matrix represented the changes of state space configurations that stabilized the control variable on the mean position (V_UCM_). The online supplements (Methods S1) show further details on partitioning variance into V_UCM_ and V_ORT_.

The ratio V_UCM_/V_ORT_ (V_RATIO_) was computed at each percentage of the normalized chair rise trajectory for each participant. If V_RATIO_ is larger than 1 for a proposed performance variable, this implies that variability in elemental variables is organized in a way to stabilize that specific performance variable around its mean across repetitions [21]. More variability in elemental variables is organized parallel to the UCM (V_UCM_) than orthogonal to the UCM (V_ORT_). Accordingly, there is a larger amount of variability which stabilizes the performance variable than variability which destabilizes the performance variable. When comparing several performance variables with each other, the performance variable of primary importance is the one with the highest V_RATIO_ [21].

### Standard deviation of joint position data

In order to relate age-related differences in flexibility of motor behaviour to kinematic adaptations in elemental variables, we also analysed the age-related differences in variability of joint position data across trials. Standard deviations of joint position data were computed at each percentage of the movement trajectory across trials and averaged across sit-to-stand phases.

### Statistical analysis

The statistical analysis of the UCM components V_UCM_, V_ORT_ and V_RATIO_ (V_UCM_/V_ORT_) focused on the lift off (31–60%) and extension phases (61–100%) of the sit-to-stand task for all performance variables.

#### Group characteristics, sit-to-stand strategies and mechafnical demands

To give a description of our groups and describe sit-to-stand strategies and mechanical demands we conducted for each of the dependent variables, BMI, the five strength measurements, sit-to-stand strategy (duration, peak trunk flexion and peak vertical GRF) and mechanical demands (peak knee and hip joint moment), a one-way ANOVA with age (young and old) as between subject factor (SPSS v. 20.0).

#### Mean phase standard deviation of performance variables

To examine mean phase standard deviations of performance variables we performed one repeated measures ANOVA on mean standard deviations of performance variables with performance variable (anterior-posterior and vertical dimension of the CoM, head, GRF and linear momentum and the angular momentum) and phase (31–60% and 61–100%) as within-subject factors and age as between-subject factor.

#### Performance variable of primary importance

To test hypotheses 1 and 2, we performed a repeated measures ANOVA on V_RATIO_ with performance variable and phase as within-subject factors and age as between-subject factor.

#### Age-related differences in flexibility of motor behaviour

To test hypothesis 3 we performed a repeated measures ANOVA on variability per DOF with UCM component (V_UCM_ and V_ORT_), dimension (anterior-posterior and vertical) and phase as within-subject factors and age as between-subject factor for the performance variable of primary importance.

#### Standard deviation of joint position data

To examine how motor flexibility is employed by the elemental variables, a repeated measures ANOVA was performed on standard deviations of joint position data with joint (foot, ankle, knee, hip, trunk, neck, elbow and shoulder) and phase as within-subject factors and age as between-subject factor.

In all of the analyses Bonferroni corrections were used to correct for multiple comparisons in post-hoc analysis. If assumptions of sphericity were violated Greenhouse-Geisser corrections were used. The level of significance was set at p<0.05.

## Results

### Group characteristics, sit-to-stand strategy and mechanical demands

Due to excessive missing values, data for one old and one young participant were excluded from the data analyses, which ultimately included 8 males and 6 females in the young group (24.3±2 years) and 6 males and 4 females in the old group (76.4±5.2). All participants performed 25 sit-to-stand trials but due to missing data, there were on average 20.3 (±2.8) and 21.2 (±3.7) trials, respectively, in the young and old group. Table 1 shows the participant’s anthropometric and strength data. The old compared to young adults’ muscle strength was lower in each muscle (p<.05).

**Table 1:** Anthropometric data and strength measurements of young and old participants.

		Young		Old	
	Muscle group	Mean	STD	Mean	STD
Age (years)		24.3	2	76.4	5.2
BMI (kg/mˆ2)*		22.6	2.7	26.8	4
Body Weight (kg)		72.2	13.2	78.7	10.5
Height (m)		1.8	.09	1.7	.07
Strength (N/kg)^a^	Hip Flexion***	3.1	0.68	1.37	0.33
	Hip Extension***	2.65	0.55	1.39	0.51
	Knee Flexion***	3.31	0.73	1.83	0.42
	Knee Extension**	5.37	1.17	3.91	0.8
	Ankle Dorsalflexion**	3.2	0.68	2.8	0.45

* indicates significant difference between age groups(* = p<.05, ** = p<.01, *** = p<.001); ^a^Strength profiles of the right leg normalized by body weight in kg.

Table 2 shows that old and young participants employed similar sit-to-stand strategies. The peak GRF was significantly lower in old compared to young adults (p<.05). The knee and hip joint moments were similar in the two groups (Table 2).

**Table 2:** Sit-to-stand strategy and mechanical demands in young and old participants.

	Young		Old	
	Mean	STD	Mean	STD
Duration (sec)	1.71	.17	1.78	.17
Trunk Flexion (°)^a^	36.95	7.98	33.69	6.73
Vertical GRF (N/kg)^b^,*	11.8	.53	11.3	.43
Peak Knee Moment (Nm/kg)^b^	2.24	.29	2.02	.25
Peak Hip Moment (Nm/kg)^b^	2.4	.36	2.21	.36

* indicates significant difference (* = p<.05); ^a^angle with the horizontal; ^b^ ground reaction forces normalized by body weight in kg and knee and ankle moments of the right leg normalized by body weight in kg.

### Joint position data

**Figure 1:**
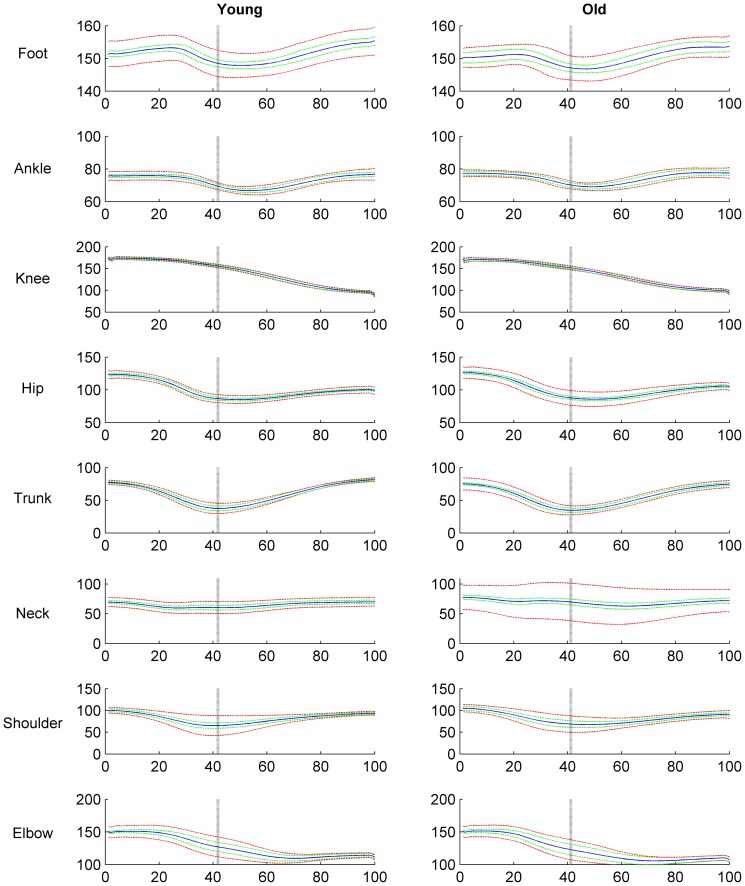
Time normalized Joint position data in young and old participants. The black line gives the mean, the dashed green line gives the standard deviation and the red line gives the standard error of the mean of the time normalized joint position data in degrees of the foot, ankle, knee, hip, trunk, neck, shoulder and elbow with the horizontal surface of the earth in young and old participants. Joint angles were calculated as depicted in figure 2 of Scholz et al 1999 [18]. The vertical dotted black line indicates time where lift-off occurred.

Figure 1 shows the joint position data for all investigated elemental variables. Joint excursions were similar between young and old adults but the hip, trunk and neck joint displayed larger deviations between the old as compared to young adults (Figure 1).

### Mean phase standard deviation of performance variables

The repeated measures ANOVA on mean phase standard deviations revealed a significant main effect for performance variable (F_1,22.3_ = 103.2, p<.001) and phase (F_1,22_ = 73.1, p<.001) and a significant two-way interaction effect between performance variable and phase (F_1,22.8_ = 62.2, p<.001). This significant interaction effect was expected regarding the kinematic and kinetic events during lift off and the extension phase of the sit-to-stand task. Interestingly, there was no significant effect for age.

### Performance variable of primary importance

**Figure 2:**
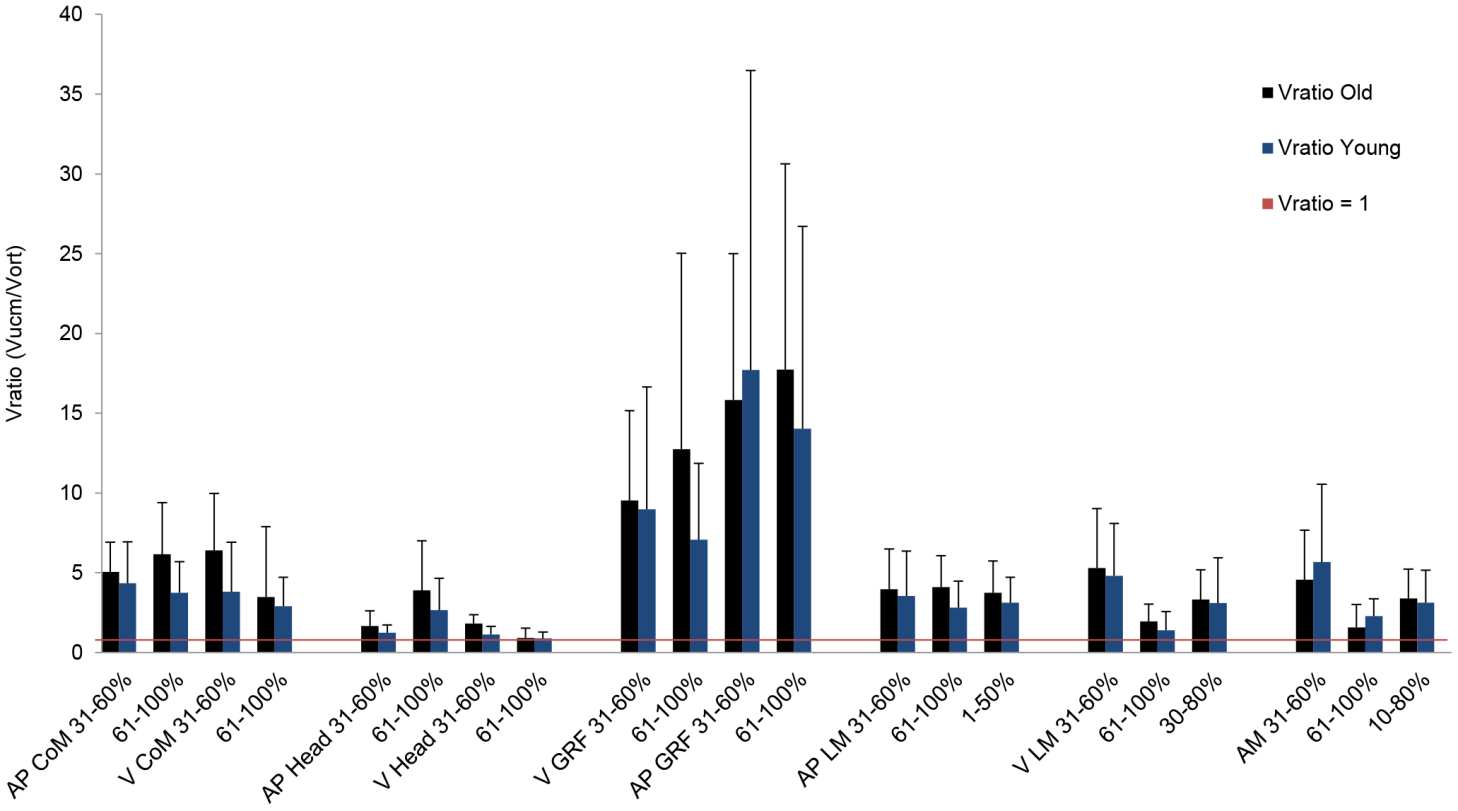
V_RATIO_ for old and young participants. V_RATIO_ of all performance variables for young and old participants in the anterior-posterior and vertical dimension of the CoM, head, GRF and linear momentum and for the angular momentum. V_RATIO_ of the CoM, head and GRF is given for lift-off (31–60%) and the extension phase (61–100%) of the chair rise. V_RATIO_ of the linear and angular momentum is given for lift-off, extension phase and the phases where greatest momentum change (0–50%; 30–80% and 10–80%) occurred. AP: anterior-posterior. V: vertical. LM: linear momentum. AM: angular momentum. Error bars represent standard error of the mean.

The repeated measures ANOVA on V_RATIO_ revealed only one significant main effect for performance variable (F_1.3,29.4_ = 25.3, p<.001). Importantly, we found no significant interaction effect between performance variable and age. Figure 2 illustrates that V_RATIO_ was largest for the anterior-posterior dimension followed by the vertical dimension of the GRF vector. These results suggest that the GRF vector was the performance variable of primary importance in both age groups. Figure 2 further shows that except for the vertical dimension of the head, all investigated performance variables displayed V_RATIOS_>1 in young and old participants during both phases of the sit-to-stand task. The results also showed a non-significant trend for old compared with young adults having higher V_RATIOs_ in all performance variables. In order to facilitate comparability with earlier studies examining CoM momentum stability with the UCM approach, Figure 2 also shows the results of the CoM momentum analysis for the phase where the greatest momentum changes occurred (1-50% for anterior-posterior, 30–80% for vertical and 10–80% for angular momentum) [17].

### Age-related differences in flexibility of motor behaviour

Table 3 shows all significant main and interaction effects from the repeated measures ANOVA on variability per DOF. There were significant main effects for age, UCM component, dimension and phase. There were significant interactions between UCM component and age, UCM component and phase, and UCM component and dimension (Table 3).

**Table 3:** Significant main and interaction effects of repeated measures ANOVA on variability per DOF.

Within-subject factor		Mean	STD	F	df	p-value
Age	Young	.0030	.0003	6.5	1,22	= .018
	Old	.0040	.0003			
UCM component	V_UCM_	.0050	.0003	168.3	1,22	<.001
	V_ORT_	.0010	.0002			
Dimension	Vertical	.0033	.0002	24.7	1,22	<.001
	Anterior-posterior	.0030	.0002			
Phase	Lift-off (31–60%)	.0040	.0003	55.8	1,22	<.001
	Extension (61–100%)	.0020	.0002			
UCM component x age				9.6	1,22	= .005
UCM component x phase				48.3	1,22	<.001
UCM component x dimension				24.7	1,22	<.001

**Figure 3:**
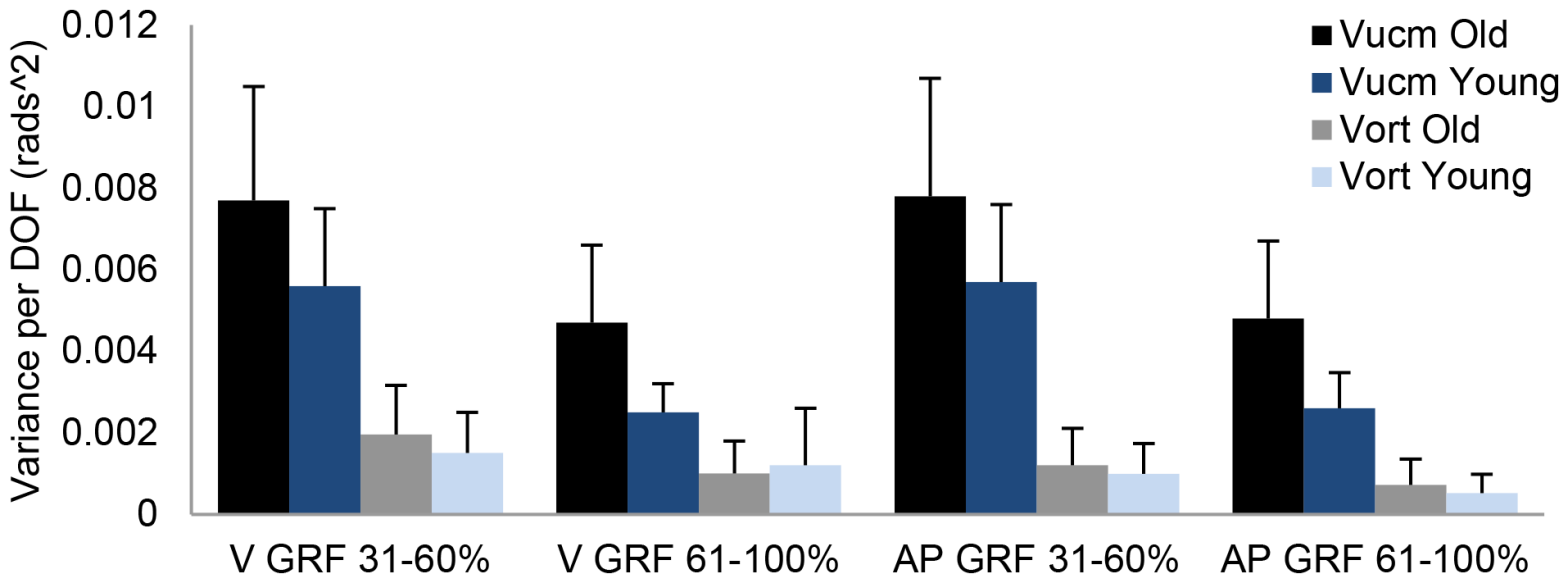
V_UCM_ and V_ORT_ for GRF in old and young participants. Variance per degree of freedom for old and young participants in the vertical and anterior-posterior dimension of the GRF during lift-off (31–60%) and extension phase (61–100%) of the sit-to-stand task. DOF: degrees of freedom. V: vertical. AP: anterior-posterior. Error bars represent standard error of the mean.

To further understand the significant interaction effect between UCM component and age we conducted two independent t-tests comparing the two age groups on V_UCM_ and V_ORT_ as post-hoc analyses. Therefore V_UCM_ and V_ORT_ were averaged across dimensions (anterior-posterior and vertical) and phases (31–60% and 61–100%). The two tests showed that V_UCM_ was significantly higher in old compared to young adults (t_12.3_ = −2.7, p = .017) and that V_ORT_ did not differ between age groups. In addition we performed post-hoc analyses of the significant interaction effect between UCM component and phase. Therefore V_UCM_ and V_ORT_ were averaged across dimensions in each phase. Two dependent t-tests compared the lift-off phase with the extension phase for V_UCM_ and V_ORT_ separately. The dependent t-tests demonstrated that V_UCM_ (t_23_ = 7.8, p<.001) and V_ORT_ (t_23_ = 4, p = .001) were significantly higher during lift-off compared to the extension phase of the sit-to-stand task. However, the mean difference between sit-to-stand phases was higher for V_UCM_ (.003±.002) compared to V_ORT_ (.0005±.006). Figure 3 provides values of V_UCM_ and V_ORT_ of the anterior-posterior and vertical dimensions for the different age groups and phases of the sit-to-stand task.

### Standard deviation of joint position data

**Figure 4:**
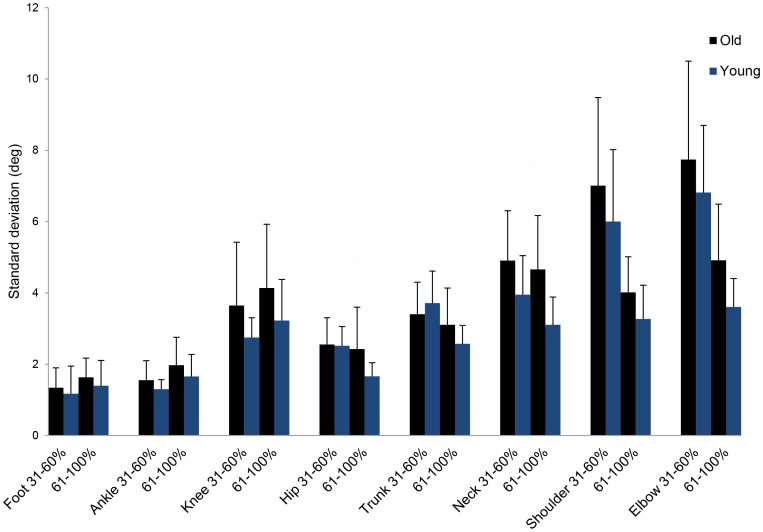
Standard deviation of joint position data in old and young participants. Across trial standard deviations for old and young adults of the foot, ankle, knee, hip, trunk, neck, shoulder and elbow joint in degrees during lift-off (31–60%) and the extension phase (61–100%) of the sit-to-stand task. Error bars represent standard error of the mean. Asterisk indicates significant difference between age groups (* = p<.05, ** = p<.01).

The repeated measures ANOVA on standard deviations revealed significant main effects for age (F_1,22_ = 8.3, p = .009), joint (F_3.8,84.3_ = 65.4, p<.001) and phase (F_1,22_ = 57.8, p<.001) and a significant two-way interaction effect between joint and phase (F = _2.79,61.4_ = 34.4, p<.001). This significant interaction effect was not unexpected regarding the different joint angle excursion during the lift-off and extension phase of the sit-to-stand task. Importantly, Figure 4 further shows that standard deviations were higher in old compared to young adults in all elemental variables during both sit-to-stand phases except for the trunk during lift-off.

## Discussion

The aim of our study was to determine the age-related differences in the performance variable of primary importance and in the use of the available motor flexibility during the sit-to-stand task. We hypothesized that young adults prioritize the control of the CoM whereas old adults prioritize the control of the GRF vector. Regarding the age-related differences in motor flexibility we explored two competing ideas: The first idea was based on previous findings and suggests that old compared to young adults employ less motor flexibility. The second idea was that because of the relatively high muscular demands of the sit-to-stand task old compared to young adults employ more motor flexibility in compensation for the strength deficits. Our main findings were that both young and old adults prioritize the stability of the GRF vector and that old adults employ more motor flexibility.

### Performance variable of primary importance

The V_RATIO_ (V_UCM_/V_ORT_) reflects to what extent the neuromuscular system stabilizes a performance variable [21]. This ratio was the highest for the anterior-posterior and vertical GRF vector in both phases of the sit-to-stand task. These results suggest that the neuromuscular system uses variability in the available degrees of freedom with the primary goal of stabilizing the GRF vector during sit-to-stand. This stabilizing effect was independent of age and muscle strength. These results are contrary to our hypothesis because we expected that young adults prioritize stability of the CoM [18], [29].

Our finding that the GRF vector is the performance variable of primary importance in old adults agrees with previous sit-to-stand studies, using different methods [6], [14]. For example, the coordinative aspects of the GRF vector during sit-to-stand explained 81% of the variance of the total time to reaching an upright position in old adults [6]. Although we derived our hypotheses based on data reporting age-related differences in neural, musculoskeletal and physiological aspects [7]–[10], [28] and previous UCM studies [18], [29], the finding that young adults prioritized stability of the GRF vector instead of the CoM trajectory was in contrast to our prediction. Because previous UCM studies did not focus on the importance of stabilizing the GRF vector, it is conceivable that they might have overlooked this aspect of neuromuscular control. In addition, GRFs are the causal factors underlying CoM movement during sit-to-stand [37]. Accordingly the trajectory of the CoM is indirectly stabilized during sit-to-stand by generating motor coordination patterns stabilizing the GRF vector. Hence our findings are not in contrast with previous literature but expand our knowledge on neuromuscular control strategies during sit-to-stand in young and old adults.

In addition to the GRF vector, our analysis showed that old and young adults’ neuromuscular system utilized variability in joint kinematics to stabilize the CoM, CoM momentum and to a lesser extent the vertical dimension of the head trajectory during both phases of the sit-to-stand task. Overall these results are in good agreement with previous literature using UCM analysis during sit-to-stand [17], [18]. However, Reisman et al (2002) reported that the vertical CoM momentum was only stabilized near the time of peak momentum [17]. We found that variability in joint angles and angular velocities were employed to stabilize the vertical CoM momentum during the entire phase where the greatest momentum change occurred (30–80%). This difference might be due to the different experimental procedures in our study compared to the study from Reisman et al (2002). In the experiment of Reisman et al (2002), participants had to perform the sit-to-stand task as fast as possible [17]. Standing up as fast as possible might have resulted in less coordinated behaviour.

The finding that several performance variables were stabilized simultaneously with the same set of elemental variables during sit-to-stand was not surprising because performance variables were functionally interlinked [23]. However, this ability of simultaneous stabilization represents an essential feature of flexible motor behaviour [17], [38]–[42] and previous experiments showed that motor abundance allows the neuromuscular system to stabilize multiple performance variables simultaneously that are functionally independent of each other [40], [42], [43].

### Age-related differences in flexibility of motor behaviour

Although young and old adults seem to prioritize stability of the same performance variable during sit-to-stand, we observed age-related differences in motor flexibility to achieve this stabilization effect. Previous studies reported that old compared to young adults’ motor behaviour is less flexible during motor-tasks [24]–[27], [30]. These findings suggest that this might also be the case during the sit-to-stand task. In contrast, we propose that the high task demands during sit-to-stand would actually necessitate the adoption of a greater degree of motor flexibility by old adults as a compensation for leg weakness. Indeed, the current results support this latter notion: old compared to young adults used a more flexible motor behaviour to stabilize the GRF vector during sit-to-stand. This greater motor flexibility in old adults was reflected by higher V_UCM_ and similar V_ORT_ compared to young adults. A greater motor flexibility implies that the neuromuscular system can choose from a greater number of motor solutions which lead to the same motor output [40]. This increase in motor flexibility improves stability of the GRF vector by enhancing the ability of the neuromuscular system to stabilize multiple performance variables simultaneously, perform a secondary task, handle new constraints or react to perturbations [17], [38]–[42]. However, as documented previously and in the present study, greater motor flexibility does not necessarily decrease variability of performance variables across trials [39].

We propose that differences in task demand between the present work and the previous studies might explain the inconsistency in results on age-related differences in motor flexibility [24]–[27], [30]. In previous studies, the relative task demands were low and similar between age groups [24]–[27], [30]. In our study old compared to young adults were significantly weaker but generated similar peak joint moments at the knee and hip during sit-to-stand. This suggests that the old adults operated at higher percentages of their maximum muscular capacity at the knee and hip. In line with this argument are the observations that rising from a chair of various heights requires 80 to 100% of the available knee extensor strength in frail and healthy old adults compared to 40–60% in healthy young adults [4], [9], [10]. Together these findings suggest that the relative task demand was likely to be more challenging for our old participants. Therefore, the larger V_UCM_, as we report it here, might be a mechanism that helps old adults to cope with the near-maximal efforts needed for successful task completion. This reasoning is in line with previous studies reporting that young adults were able to stabilize task-important performance variables to a similar extent by increasing the amount of V_UCM_ and leaving V_ORT_ unchanged when they rose from a chair under mechanically more challenging conditions [16], [17]. Thus, freeing those DOF that stabilize task-important performance variables seems to be a strategy used by the aging neuromuscular system to compensate for strength deficits at the knee and hip. This strategy is reflected in higher V_RATIO_ and higher V_UCM_ values for old compared to young adults in a sit-to-stand task, and such strategies might be less necessary in less demanding tasks. In line with this reasoning, recent studies on the effect of fatigue on motor flexibility in multi-finger force production tasks have shown that with increasing fatigue in one finger, V_UCM_ increases and V_ORT_ remains the same with a concomitant preservation of task accuracy [44], [45].

Our interpretation of the data rises the question what kinematic adaptations were associated with an increase in motor flexibility. Theoretically, any increase in variability of a restricted set of joint angles or all joint angles could mediate increases in V_UCM_. Considering the fairly constrained nature of the sit-to-stand task, kinematic adjustments would most likely occur at the trunk, shoulder or elbow joint. However, standard deviations of joint position data revealed that old compared to young adults employed larger amounts of variability in all elemental variables during both phases of the sit-to-stand task except for the trunk during lift-off. Because V_ORT_ did not differ between the two groups, it is likely that the increase in old adults’ joint position variability was selectively directed into variability stabilizing the GRF vector (V_UCM_). Hence, the aging neuromuscular system makes use of motor abundance and employs larger amounts of joint position variability in all elemental variables to improve stability of the GRF vector during sit-to-stand.

Considering that an increase in V_UCM_ seems to have exclusively favourable benefits for the neuromuscular system in old adults, the question arises whether interventions could exploit these benefits. It is reasonable to assume that there is a finite amount of V_UCM_ old adults can access during the execution of motor tasks. When older adults with strength deficits employ large amounts of V_UCM_ during a routine sit-to-stand task, they would operate closer to their maximal available motor flexibility compared to their young counterparts. Operating closer to the maximal available motor flexibility would diminish the reserve capacity of the neuromuscular system which in turn might impair old adults’ ability to adequately react to perturbations, perform a secondary task or handle new constraints[17], [38]–[42]. Following this line of reasoning we propose that next to traditional strength training, intervention and prevention programs should consider incorporating exercises based on idea that training of a large variety of between-exercise differences facilitates the exploitation of numerous possible motor solutions. The exploitation of possible motor solutions would increase the amount of available motor coordination patterns from which the aging neuromuscular system can choose to achieve successful sit-to-stand. In the literature this idea has been proposed in different views on motor learning. It is known as variability of practice[46]–[48] but can also be found in the differential learning concept [49], [50]. For the sit-to-stand task this would imply that standing up could be executed from different chair heights, support surfaces or start and end postures, which would result in a larger range of practiced motor coordination patterns. An increase in the available motor coordination patterns would increase the old adults’ available motor flexibility and improve the ability to accurately and stable perform the sit-to-stand task under a variety of performance contexts [17], [38]–[42].

### Limitations and future research

The present study has several limitations. First, the analysis of the sit-to-stand task focused on the sagittal plane. Therefore, the analysis did not include variability in elemental variables in the frontal plane, which might have affected the stability of the investigated performance variables. In addition, undetected and unquantified deficits in balance control might have made the sit-to-stand task even more challenging for the old participants. Finally, in this initial effort we did not manipulate task difficulty to determine systematically the nature of how exactly the increase in motor flexibility, as quantified by UCM analysis, acted as a compensatory mechanism for old adults’ lower extremity weakness. Future research might aim to systematically investigate to which extent flexibility in motor behaviour changes in young and old adults with increasing demands on the muscular and balance control system and how improvements in leg strength affect flexibility of the motor behaviour in old adults.

## Conclusions

We found that age affects motor coordination strategies during sit-to-stand. Independent of age and muscle strength both old and young adults prioritized stability of the GRF vector when rising from a chair. However old differed from young adults in the flexibility of their motor behaviour. Old adults employed a more flexible motor behaviour during sit-to-stand as compared to young adults. Due to the higher demand of the sit-to-stand task in the old participants we propose that freeing those degrees of freedom stabilizing task-important performance variables is a strategy used by the aging neuromuscular system to compensate for strength deficits and improve stability. These findings might have implications for the design of interventions, which aim to prolong old adults’ motor independence.

## Supporting Information

Methods S1 **Provides additional information on the geometric model of the CoM, computation of the Jacobian with Multiple Linear Regression Analysis (MLR), partitioning variance into V_UCM_ and V_ORT_, and the statistical analysis comparing the MLR approach with the geometric model approach.**
(DOCX)

Results S1 **Gives the results from the one-way ANOVA on variability per DOF with V_UCM_ and V_ORT_ as dependent and the computational approach (MLR and geometric model) as independent variable.**
(DOCX)

Discussion S1 **Provides a brief discussion on the analysis whether MLR is a valid method to calculate the Jacobian for the anterior-posterior CoM position in an eight-DOF system.**
(DOCX)
