# Supplementary material for: Not All Is Lost: Old Adults Retain Flexibility in Motor Behaviour during Sit-to-Stand
Source: PLoS One. 2013 Oct 25;8(10):e77760. doi: 10.1371/journal.pone.0077760 (PMC3808394; doi:10.1371/journal.pone.0077760)
Supplement: Methods S1 — Provides additional information on the geometric model of the CoM, computation of the Jacobian with Multiple Linear Regression Analysis (MLR), partitioning variance into VUCM and VORT, and the statistical analysis comparing the MLR approach with the geometric model approach. (DOCX) [file pone.0077760.s001.docx]

**Methods S1**

***UCM analysis***

Geometric model of the CoM

The joint configuration formed between the foot, shank, thigh, pelvis, trunk, head/neck, forearm, and arm segments with the horizontal surface of the earth were used in the estimation procedure to simplify the model equations. Correspondingly, there were 8 dimensions of the joint configuration space with regard to the hypothesis of the CoM control. The geometric model of the CoM is given by equations 1 and 2 [[1](#_ENREF_1)]:

$$\begin{matrix} \text{CoMx = [0.0145 * lft * cos}\theta\text{ft + 0.093 * lft * cos}\theta\text{ft + 0.05273 * lsh * cos}\theta\text{sh + 0.2 * } \\ \text{(lft * cos}\theta\text{ft + lsh * cos}\theta\text{sh) +0.113 * lth * cos}\theta\text{ft + 0.0142 * (lft * cos}\theta\text{ft + lsh * } \\ \text{cos}\theta\text{sh + lh * cos}\theta\text{th) + 0.0149 * lpv * cos}\theta\text{pv +0.355 * (lft * cos}\theta\text{ft + lsh * cos}\theta\text{sh + } \\ \text{lth * cos}\theta\text{th + lpv *cos}\theta\text{pv) + 0.2237 * ltr * cos}\theta\text{tr +...0.181 (lft * cos}\theta\text{ft + lsh * } \\ \text{cos}\theta\text{sh + lth * cos}\theta\text{th + lpv * cos}\theta\text{pv + ltr * cos}\theta\text{tr + lhn * cos}\theta\text{hn) +0.056 * (lft * } \\ \text{cos}\theta\text{ft + lsh * cos}\theta\text{sh + lth * cos}\theta\text{th + lpv * cos}\theta\text{pv + ltr * cos}\theta\text{tr) + 0.0244 lar * } \\ \text{cos}\theta\text{ar +0.044 * (lft * cos}\theta\text{ft + lsh * cos}\theta\text{sh + lth * cos}\theta\text{th + lpv * cos}\theta\text{pv + ltr * } \\ \text{cos}\theta\text{tr + lar * cos}\theta\text{ar) +0.030 * lfa * cos}\theta\text{fa ] / Mtot} \\ \end{matrix}$$

$$\begin{matrix} \text{CoMy = [0.0145 * lft * sin}\theta\text{ft + 0.093 * lft * sin}\theta\text{ft + 0.05273 * lsh * sin}\theta\text{sh + 0.2 * } \\ \text{(lft * sin}\theta\text{ft + lsh * sin}\theta\text{sh) +0.113 * lth * sin}\theta\text{ft + 0.0142 * (lft * sin}\theta\text{ft + lsh * } \\ \text{sin}\theta\text{sh + lh * sin}\theta\text{th) + 0.0149 * lpv * sin}\theta\text{pv +0.355 * (lft * sin}\theta\text{ft + lsh * sin}\theta\text{sh + } \\ \text{lth * sin}\theta\text{th + lpv *sin}\theta\text{pv) + 0.2237 * ltr * sin}\theta\text{tr +...0.181 (lft * sin}\theta\text{ft + lsh * } \\ \text{sin}\theta\text{sh + lth * sin}\theta\text{th + lpv * sin}\theta\text{pv + ltr * sin}\theta\text{tr + lhn * sin}\theta\text{hn) +0.056 * (lft * } \\ \text{sin}\theta\text{ft + lsh * sin}\theta\text{sh + lth * sin}\theta\text{th + lpv * sin}\theta\text{pv + ltr * sin}\theta\text{tr) + 0.0244 lar *} \\ \text{sin}\theta\text{ar +0.044 * (lft * sin}\theta\text{ft + lsh * sin}\theta\text{sh + lth * sin}\theta\text{th + lpv * sin}\theta\text{pv + ltr * } \\ \text{sin}\theta\text{tr + lar * sin}\theta\text{ar) +0.030 * lfa * sin}\theta\text{fa ] / Mtot} \end{matrix}$$

Where $CoMx$ is the sagittal plane CoM position in x-coordinates, $CoMy$ the sagittal plane CoM position in y-coordinates, ft= foot, sh = shank, th = thigh, pv = pelvis, tr = trunk, hn = head/neck, fa = forearm, pv= pelvis,$\theta$i are segment angles, and li is the length of body segment i. The constant values denote the product of a segment’s mass as a proportion of total body mass times its location from the proximal joint, as a proportion of the total segment length. Mtot represents the total body mass. The trunk was assumed, to form one rigid segment between the 5^th^ lumbar and 7^th^ cervical vertebrae, while the pelvis was defined as the segment from the hip joint to the 5^th^ lumbar vertebra.

Jacobian with Multiple Linear Regression Analysis

The Jacobian was obtained from the coefficients of the MLR analysis at each percentage of the time-normalized chair rise trajectory across repetitions. The difference between joint angle θ_ij_ – mean(θ_j_) was the independent variable, where i is the percentage of the movement trajectory and j is the repetition calculated for all angles (foot, ankle, knee, thigh, trunk, upper arm, lower arm and neck). The difference between anterior-posterior CoM_ij_ – mean(CoM_i_) was the dependent variable. Accordingly θ and dθ/dt were used as independent variables for the CoM momentum calculations. Further UCM analysis was performed as described below.

Partitioning Variance into V_UCM_ and V_ORT_

In order to calculate changes in joint configuration patterns affecting the position of the CoM, variability per degree of freedom (DOF) was computed. The component of variability perpendicular to the null space (V_ORT_) was calculated by equation 3 [[2](#_ENREF_2)]:

1.

Where D_variable_ is the dimension of the performance variable. Accordingly, the component of variability stabilizing the performance variable is computed by equation 4 [[2](#_ENREF_2)]:

1.

***Statistical analysis***

Whether MLR is a valid method for computing the Jacobian in an eight DOF system was investigated by performing a one-way ANOVA on variability per DOF with V_UCM_ and V_ORT_ as dependent and the computational approach (MLR and geometric model) as independent variable.

**References**

1. Scholz JP, Schöner G (1999) The uncontrolled manifold concept: identifying control variables for a functional task. Exp Brain Res 126: 289-306.

2. Van der Steen MC, Bongers RM (2011) Joint angle variability and co-variation in a reaching with a rod task. 208: 411-422.
