# Supplementary material for: Not All Is Lost: Old Adults Retain Flexibility in Motor Behaviour during Sit-to-Stand
Source: PLoS One. 2013 Oct 25;8(10):e77760. doi: 10.1371/journal.pone.0077760 (PMC3808394; doi:10.1371/journal.pone.0077760)
Supplement: Results S1 — Gives the results from the one-way ANOVA on variability per DOF with VUCM and VORT as dependent and the computational approach (MLR and geometric model) as independent variable. (DOCX) [file pone.0077760.s002.docx]

**Results S1**.

***MLR and geometrical model approach***

The one-way ANOVA on variability per DOF revealed that there was no significant difference in the outcomes of the UCM analysis calculated by the MLR and geometric model approach.
