# Supplementary material for: Not All Is Lost: Old Adults Retain Flexibility in Motor Behaviour during Sit-to-Stand
Source: PLoS One. 2013 Oct 25;8(10):e77760. doi: 10.1371/journal.pone.0077760 (PMC3808394; doi:10.1371/journal.pone.0077760)
Supplement: Discussion S1 — Provides a brief discussion on the analysis whether MLR is a valid method to calculate the Jacobian for the anterior-posterior CoM position in an eight-DOF system. (DOCX) [file pone.0077760.s003.docx]

**Discussion S1**

***MLR vs Geometric model***

This supplement addressed whether MLR analysis was a valid method to calculate the Jacobian for the anterior-posterior CoM position in an eight-DOF system. Results showed a non-significant difference in the outcomes of the UCM analysis calculated by the MLR and geometric model approach. These results were similar to those of Freitas et al (2010) [[1](#_ENREF_1)]. Based on our results and those from Freitas et al (2010) it was assumed that the MLR analysis is a valid method for computing the Jacobian and was used for further UCM analysis. Note that no further validation of the MLR approach was performed for the UCM analysis of other investigated performance variables.

**References**

1. de Freitas SM, Scholz JP, Latash ML (2010) Analyses of joint variance related to voluntary whole-body movements performed in standing. J Neurosci Methods 188: 89-96.
